# Slope classicality in higher Coleman theory via highest weight vectors in completed cohomology

**DOI:** 10.1073/pnas.2208249119

**Published:** 2022-11-02

**Authors:** Sean Howe

**Affiliations:** ^a^Department of Mathematics, University of Utah, Salt Lake City, UT 84112

**Keywords:** *p*-adic and overconvergent modular forms, completed cohomology, higher Coleman theory, classicality and control theorems

## Abstract

We give a proof of the slope classicality theorem in classical and higher Coleman theory for modular curves of arbitrary level using the completed cohomology classes attached to overconvergent modular forms. The latter give an embedding of the quotient of overconvergent modular forms by classical modular forms, which is the obstruction space for classicality in either cohomological degree, into a unitary representation of ${\text{GL}}_{2}({\mathbb{Q}}_{p})$. The *U_p_* operator becomes a double coset, and unitarity yields slope vanishing.

Fix a sufficiently small compact open subgroup $K^{p}\leq {\text{GL}}_{2}(A_{f}^{\left(p\right)})$ and let ${\mathbb{C}}_{p}$ be the completion of an algebraic closure of ${\mathbb{Q}}_{p}$. Let $X_{1}(p^{n})/{\mathbb{C}}_{p}$ be the smooth compactification of the modular curve parameterizing elliptic curves with a point of exact order *p^n^* and level *K^p^* structure. Everywhere below, we view $X_{1}(p^{n})$ as an adic space over ${\mathbb{C}}_{p}$. The closed canonical ordinary locus $X_{1}{\left(p^{n}\right)}_{e}$ is the topological closure of the locus of rank one points parameterizing elliptic curves of ordinary reduction equipped with a point generating the canonical subgroup of level *p^n^*. We write $X_{1}{\left(p^{n}\right)}_{w}=X_{1}(p^{n})\backslash X_{1}{\left(p^{n}\right)}_{e}$ for its open complement (the subscripts *e* and *w* refer to the trivial and nontrivial elements of the Weyl group for ${\text{GL}}_{2}$).

Writing *ω* for the modular sheaf, the space $H^{0}(X_{1}{\left(p^{n}\right)}_{e},\omega^{k})$ is naturally identified with the direct sum of spaces of overconvergent modular forms of weights *κ* such that $\kappa =z^{k}\chi$ where *χ* is a character of ${\left(\mathbb{Z}/p^{n}\mathbb{Z}\right)}^{\times }$. From the perspective of the higher Coleman theory of Boxer and Pilloni (1, 2), it is natural to also consider the compactly supported cohomology $H_{c}^{1}(X_{1}{\left(p^{n}\right)}_{w},\omega^{k})$. These groups are related by the exact sequence of compactly supported topological sheaf cohomology[1]
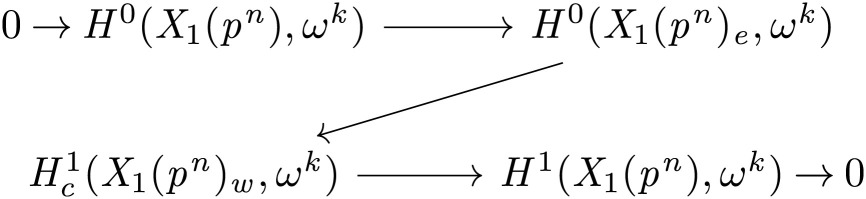


arising from the following exact sequence of sheaves on the topological space $X_{1}(p^{n})$ obtained via push–pull of *ω^k^* along the inclusions $j:X_{1}{\left(p^{n}\right)}_{w},\to X_{1}(p^{n})$ and $i:X_{1}{\left(p^{n}\right)}_{e},\to X_{1}(p^{n})$:

$$0\to j_{!}j^{-1}\omega^{k}\to \omega^{k}\to i_{*}i^{-1}\omega^{k}\to 0.$$

As in refs. 1 and 2 (see also section 2.2 below), there is an operator *U_p_* on each of these spaces induced by a cohomological correspondence and extending a classical double-coset Hecke operator *U_p_* on $H^{•}(X_{1}(p^{n}),\omega^{k})$ (up to matching choices of the normalization). For any s∈R and ${\mathbb{C}}_{p}$ vector space *V* equipped with an action of a linear operator *U_p_*, we can pass to the part $V^{<s}$ of slope less than *s*, defined to be the span of all generalized eigenspaces of *U_p_* for eigenvalues *λ* with $|\lambda |>p^{-s}$.

Theorem 1. *For* t∈ℤ\{0}, Eq. **1** *induces isomorphisms*

$$\begin{array}{ll} H^{0}{\left(X_{1}(p^{n}),\omega^{1+t}\right)}^{<|t|} & =H^{0}{\left(X_{1}{\left(p^{n}\right)}_{e},\omega^{1+t}\right)}^{<|t|}\;\;\text{and}\;\\ H_{c}^{1}{\left(X_{1}{\left(p^{n}\right)}_{w},\omega^{1+t}\right)}^{<|t|} & =H^{1}{\left(X_{1}(p^{n}),\omega^{1+t}\right)}^{<|t|}. \end{array}$$

In cohomological degree zero, this is a result of Coleman (3, 4). In degree one, this is a result of Boxer and Pilloni (1, 2) (who also reprove Coleman’s result).* We give a short proof using the connection between overconvergent modular forms and the completed cohomology of modular curves established in refs. 5 and 6. This provides a perspective on a fundamental result in the *p*-adic theory of automorphic forms: We recall that Coleman’s proof (in the degree zero case) is based on an analysis of the de Rham cohomology of modular curves and a clever dimension counting, while the proof of Boxer and Pilloni is based on slope estimates established via an analysis of cohomological correspondences and integral structures on coherent cohomology. Our proof, by contrast, proceeds by embedding the defect to classicality in completed cohomology so that the necessary slope estimates are a trivial consequence of unitarity, itself a trivial consequence of the construction of completed cohomology from integral singular cohomology (see Remark 1 for the origins of this approach in Emerton’s classicality for Jacquet modules). This depends on strong nondegeneracy results of refs. 5 or 6, but the actual construction of the cohomology classes is completely explicit, so that our proof of Theorem 1 reduces to elementary matrix computations.

## 1. Proof of Theorem 1

Theorem 1 is an immediate consequence of Lemma 1 below, itself an immediate consequence of the results of refs. 5 or 6.

Let *X* be the infinite-level (compactified) modular curve of prime-to-*p*-level *K^p^*. It admits an action of ${\text{GL}}_{2}({\mathbb{Q}}_{p})$ and, by Scholze’s primitive comparison (see ref. 6, corollary 4.4.3), $H^{1}(X,O_{X})$ is identified with the ${\mathbb{C}}_{p}$-completed cohomology of the tower of modular curves of prime-to-*p*-level *K^p^*. We need only that it is a Banach space with a unitary action of ${\text{GL}}_{2}({\mathbb{Q}}_{p})$, which follows because the unit ball, that is, the image of $H^{1}(X,O_{X}^{+})$, is preserved by ${\text{GL}}_{2}({\mathbb{Q}}_{p})$.

Lemma 1. *Let U_p_ denote the Hecke operator of refs.* 1 *and* 2 (*the normalization depends on the weight; see* *section* 2), *and let* $N\leq {\text{GL}}_{2}$ *be the group of upper triangular unipotent matrices. For* t≠0, *the cup products of ref.* 5 *give an embedding*

$$H^{0}(X_{1}{\left(p^{n}\right)}_{e},\omega^{1+t})/H^{0}(X_{1}(p^{n}),\omega^{1+t})\;,\to \;H^{1}{\left(X,O_{X}\right)}^{N({\mathbb{Z}}_{p})}$$

*matching* $U_{p}$ *with the double coset* $p^{\left|t\right|}\cdot [N({\mathbb{Z}}_{p})\text{diag}(p,1)N({\mathbb{Z}}_{p})].$

Proof of Theorem 1 , assuming Lemma 1: Consider

$$p^{\left|t\right|}\cdot [N({\mathbb{Z}}_{p})\text{diag}(p,1)N({\mathbb{Z}}_{p})]=p^{\left|t\right|}\sum_{i=0}^{p-1}\left[\begin{array}{cc} p & i\\ 0 & 1 \end{array}\right]$$

acting on $H^{1}{\left(X,O_{X}\right)}^{N({\mathbb{Z}}_{p})}$. Because the action of ${\text{GL}}_{2}({\mathbb{Q}}_{p})$ is unitary, it has operator norm $\leq 1/p^{\left|t\right|}$, so the slope <|t| part vanishes in $H^{1}{\left(X,O_{X}\right)}^{N({\mathbb{Z}}_{p})}$. If we write

$$Q_{t}≔H^{0}(X_{1}{\left(p^{n}\right)}_{e},\omega^{1+t})/H^{0}(X_{1}(p^{n}),\omega^{1+t}),$$

then, combining the above with Lemma 1, we find that, for $t\neq 0,\;Q_{t}^{<|t|}=0$. To obtain Theorem 1, we split Eq. **1** for k=1+t into two short exact sequences,

$$\begin{array}{ll} & 0\;\to \;H^{0}(X_{1}(p^{n}),\omega^{1+t})\;\to \;H^{0}(X_{1}{\left(p^{n}\right)}_{e},\omega^{1+t})\;\to \;Q_{t}\;\to \;0\;\text{and}\\ & 0\;\to \;Q_{t}\;\to \;H_{c}^{1}(X_{1}{\left(p^{n}\right)}_{w},\omega^{1+t})\;\to \;H^{1}(X_{1}(p^{n}),\omega^{1+t})\;\to \;0. \end{array}$$

Taking the slope <|t| part yields the isomorphisms—for the first sequence this is immediate, since this functor is always left exact, and, for the second sequence, right exactness follows from compactness of the *U_p_* operator on overconvergent forms. □

Remark 1. As recalled in Remark 3 below, for *t*  >  0, the embedding of Lemma 1 arises from a highest weight vector in the irreducible submodule of a Verma module with algebraic quotient. The argument is then essentially the same as Emerton’s proof of the classicality theorem for locally analytic Jacquet modules (ref.  7, theorem 4.4.5).

It thus remains only to prove Lemma 1. This is essentially immediate from the results of refs. 5 or 6, once the ${\text{GL}}_{2}({\mathbb{Q}}_{p})$ actions are matched up. This matching is actually a bit subtle, as there are multiple possible conventions for the Hodge–Tate period map and the equivariant structure on the modular sheaf. Any set of choices gives the same ${\text{GL}}_{2}({\mathbb{Q}}_{p})$-action modulo inverse transpose and some determinants, so, often, the precise choices are irrelevant. Here, however, we must follow a power of *p* coming from the action of diag(p,1), so it is crucial to screw our heads on exactly right on this point. In the next section, we fix normalizations, then prove Lemma 1.

## 2. Normalizations and Proof of Lemma 1

### 2.1. Choices.

We fix the action of ${\text{GL}}_{2}({\mathbb{Q}}_{p})$ on *X* so that, over the noncompactified infinite-level curve *Y*, ${\text{GL}}_{2}({\mathbb{Q}}_{p})=\text{Aut}({\mathbb{Q}}_{p}^{2})$ acts by composition with the trivialization of the Tate module of the universal elliptic curve; that is, we use the action on the homological normalization of the moduli problem. This differs by an inverse transpose from the cohomological normalization, where the action is on the trivialization of the first étale cohomology of the universal elliptic curve.

We take the Hodge–Tate period map $\pi_{\text{HT}}:X\to {\mathbb{P}}^{1}$ so that $\pi_{\text{HT}}{|}_{Y}$ is the classifying map for the Hodge–Tate line inside of the first étale cohomology of the universal elliptic curve. Thus, over *Y*, we have a ${\text{GL}}_{2}({\mathbb{Q}}_{p})$-equivariant commuting diagram,


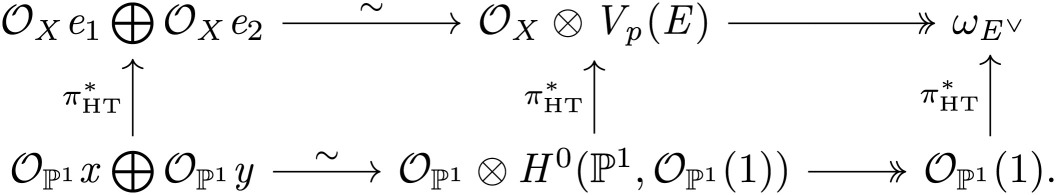


Here *e*_1_ and *e*_2_ are the universal basis for the Tate module $V_{p}(E)=T_{p}(E)[1/p]$, *x* and *y* are the standard basis for $H^{0}({\mathbb{P}}^{1},O_{{\mathbb{P}}^{1}}(1))$ so that homogeneous coordinates are [x:y], and $E^{\vee }$ denotes the dual of the universal elliptic curve. Of course, there is a canonical isomorphism $E^{\vee }≅E$ inducing $\omega_{E^{\vee }}≅\omega_{E}$; however, this isomorphism does not respect the natural ${\text{GL}}_{2}({\mathbb{Q}}_{p})$-equivariant structures! Equivariantly,

$$\omega_{E^{\vee }}=\omega_{E}⊗{\left|\det\right|}^{-1},$$

where, here, |det| comes from the action of isogenies on $H^{1}(E,\Omega_{E})$. Note that this twist is actually on the entire ${\text{GL}}_{2}(A_{f})$ action (via ${\left(g_{\ell }\right)}_{\ell }\mapsto \prod_{\ell }|\det\;(g_{\ell }){|}_{\ell }^{-1}$), so that the distinction between these equivariant structures also determines the normalization of the prime-to-*p* Hecke operators. Below we will continue, as in the introduction, to write simply *ω* for the modular sheaf, with the understanding that we have adopted the equivariant structure described above.

Under the natural map to $X\to X_{1}(p^{n}),\;X_{1}{\left(p^{n}\right)}_{e}$ is the image of $\pi_{\text{HT}}^{-1}([0:1])$, and the action of ${\text{GL}}_{2}({\mathbb{Q}}_{p})$ on $\langle x,y\rangle =H^{0}({\mathbb{P}}^{1},O_{{\mathbb{P}}^{1}}(1))$ is by the standard representation

$$\left[\begin{array}{cc} a & b\\ c & d \end{array}\right]\cdot x=ax+cy\;\text{and}\;\left[\begin{array}{cc} a & b\\ c & d \end{array}\right]\cdot y=bx+dy.$$

### 2.2. The $U_{p}^{\text{naive}}$ Operator.

The operator $U_{p}^{\text{naive}}$ at level $X_{1}(p^{n})$ of refs. 1 and 2 is defined using the correspondence *C* parameterizing degree *p* isogenies $\psi :(E_{1},P_{1})\to (E_{2},P_{2})$ (here we suppress prime-to-*p*-level structure from the notation). Writing the two obvious projections as $p_{1},p_{2}:C\to X_{1}(p^{n}),\;U_{p}^{\text{naive}}$ is defined on *ω^k^* as $\text{tr}{}^{\circ}p_{1}{}_{!}{}^{\circ}\psi^{*}{}^{\circ}p_{2}^{*}$. Given a geometric point (E,P) that is not a cusp and a nonzero differential *η* on *E*, we can compute $\left(U_{p}^{\text{naive}}f)(E,P,\eta \right)$ as follows: First, choose a basis $\left(e_{1},e_{2}\right)$ of $T_{p}(E)$ such that *e*_1_ reduces to *P* mod *p^n^*. Then, for 0≤i≤p−1, write

$$\psi_{i}:E\to E_{i}≔E/\langle i{\bar{e}}_{1}+{\bar{e}}_{2}\rangle ,$$

where ${\bar{e}}_{i}$ denotes the image of *e_i_* in E[p]. Then $\psi_{i}^{*}:\omega_{E_{i}}\to \omega_{E}$ is invertible, and

[2] $$(U_{p}^{\text{naive}}f)(E,P,\eta )=\sum_{i=0}^{p-1}f(E_{i},\psi_{i}(P),{\left(\psi_{i}^{*}\right)}^{-1}\eta ).$$

We will now realize this same $U_{p}^{\text{naive}}$ as a double-coset operator: Let *B* denote the upper triangular Borel in ${\text{GL}}_{2}$. The space of overconvergent modular forms of weight *k* at any finite level $\Gamma_{1}(p^{n})$ is naturally embedded as the $B_{1}(p^{n})=\Gamma_{1}(p^{n})\cap B({\mathbb{Q}}_{p})$ invariants in the $B({\mathbb{Q}}_{p})$ representation

$$M_{k}^{†}≔H^{0}([0:1],{\left(\pi_{\text{HT}}{}_{*}\pi_{\text{HT}}^{*}O(k)\right)}^{\text{sm}}),$$

where, here, the superscript sm denotes the subsheaf of $\pi_{\text{HT}}{}_{*}\pi_{\text{HT}}^{*}O(k)=\pi_{\text{HT}}{}_{*}\omega^{k}$ whose sections over a quasi-compact open *V* are those with locally constant orbit map for the action of the stabilizer of *V* in ${\text{GL}}_{2}({\mathbb{Q}}_{p})$ (i.e., sections fixed by some compact open subgroup of ${\text{GL}}_{2}({\mathbb{Q}}_{p})$, i.e., sections coming from finite level). For more on this construction, see ref. 5, section 3.1.

The space $M_{k}^{†}$ contains the space of classical modular forms

$$M_{k}^{\text{cl}}=H^{0}({\mathbb{P}}^{1},{\left(\pi_{\text{HT}}{}_{*}\pi_{\text{HT}}^{*}O(k)\right)}^{\text{sm}})$$

$B({\mathbb{Q}}_{p})$ equivariantly by restriction. The action of

$$U≔N({\mathbb{Z}}_{p})\text{diag}(p,1)N({\mathbb{Z}}_{p})=\sum_{i=0}^{p-1}\left[\begin{array}{cc} p & i\\ 0 & 1 \end{array}\right]≕\sum_{i=0}^{p-1}A_{i}$$

on $M_{k}^{†,N({\mathbb{Z}}_{p})}$ is identified with $U_{p}^{\text{naive}}$ on $M_{k}^{†,B_{1}(p^{n})}=H^{0}(X_{1}(p^{n}),\omega^{k})$—we explain this computation now. It suffices to check at geometric points away from cusps, so we can compare with the explicit formula of Eq. **2** for $U_{p}^{\text{naive}}$. Now,

$$\begin{array}{rl} U(E,e_{1},e_{2},\eta ) & =\sum_{i=0}^{p-1}\left(A_{i}\cdot f\right)(E,e_{1},e_{2},\eta )\\ & =\sum_{i=0}^{p-1}f(E,pe_{1},ie_{1}+e_{2},\eta ), \end{array}$$

and, by the commuting diagram


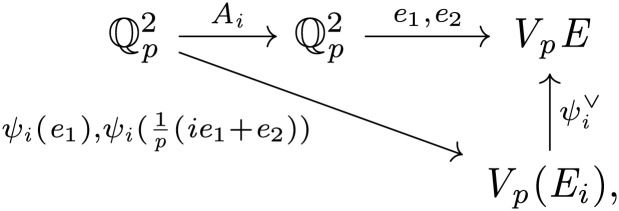


we see that $f(E,pe_{1},ie_{1}+e_{2},\eta )$ is equal to $f(E_{i},\psi_{i}(e_{1}),\psi_{i}(\frac{1}{p}(ie_{1}+e_{2})),\eta_{i})$ for some *η_i_*. One might guess that *η_i_* is ${\left(\psi_{i}^{\vee }\right)}^{*}\eta$, but it is not! This would hold if we used the *ω_E_*-equivariant structure, but, in the $\omega_{E^{\vee }}$ equivariant structure, we must replace $\psi_{i}^{\vee }$ with its dual *ψ_i_* so that $\eta_{i}={\left(\psi_{i}^{*}\right)}^{-1}\eta$. Thus we recover the right-hand side of Eq. **2**.

### 2.3. Proof of Lemma 1 and Concluding Remarks.

Recall (ref. 1, sentence preceding theorem 5.13) the normalized operator

$$U_{p}≔\{\begin{array}{ll} p^{-1}U_{p}^{\text{naive}} & \text{if}\;k\geq 1\\ p^{-k}U_{p}^{\text{naive}} & \text{if}\;k\leq 1 \end{array}\;\text{so}\;U_{p}^{\text{naive}}=\{\begin{array}{ll} pU_{p} & \text{if}\;k\geq 1\\ p^{k}U_{p} & \text{if}\;k\leq 1. \end{array}$$

appearing in the statement of Lemma 1 (see also Remark 4).

Proof of Lemma 1. We first treat the case k=1+t≥2. Then, for any $s\in M_{k}^{†},\;s/x^{k}$ is a section of $O_{X}$ defined on the preimage of a punctured neighborhood of [0:1] under $\pi_{\text{HT}}$. It thus determines a Cech cohomology class $\left[s/x^{k}\right]$ in $H^{1}(X,O_{X})$. Then, ref. 5, theorem A implies that $s\mapsto [s/x^{k}]$ induces an injection $M_{k}^{†}/M_{k}^{\text{cl}},\to H^{1}(X,O_{X})$; actually, in ref. 5, the results are stated using cusp forms and compactly supported completed cohomology, but, given the identification of $H^{1}(X,O_{X})$ with completed cohomology, one obtains the desired statement by the same arguments. The map is $B({\mathbb{Q}}_{p})$ equivariant if one twists the action on $M_{k}^{†}$ by

$$\left[\begin{array}{cc} a & b\\ 0 & c \end{array}\right]\mapsto a^{-k}$$

(the twist comes from the action on *x^k^*, of course!). We deduce that $p^{-k}U_{p}^{\text{naive}}=p^{-k}(pU_{p})=p^{-t}U_{p}$ is identified with $\left[N({\mathbb{Z}}_{p})\text{diag}(p,1)N({\mathbb{Z}}_{p})\right]$, as desired.

We now treat the case k=1+t≤0. In this case, ref. 5, theorem A shows that $s\mapsto [s/(xy^{t})]$ induces an embedding $M_{k}^{†}/M_{k}^{\text{cl}},\to H^{1}(X,O_{X})$. Actually, here one must be slightly more careful invoking the arguments of ref. 5, which are stated with cusp forms, in the case *k* = 0: Of course, $M_{k}^{\text{cl}}=0$ when *k*  <  0, but, when *k* = 0, we have that $M_{0}^{\text{cl}}$ is the locally constant functions, whereas the cusp forms are still trivial. However, it is elementary to see that $M_{0}^{\text{cl}}$ is in the kernel (as $s/(xy^{-1})=s/z$ extends to a function on the complement of [0:1] where 1/z is a local coordinate), and the argument of *loco citato* still establishes an injection on the quotient by $M_{0}^{\text{cl}}$. The embedding is $B({\mathbb{Q}}_{p})$ equivariant if we twist $M_{k}^{†}$ by

$$\left[\begin{array}{cc} a & b\\ 0 & c \end{array}\right]\mapsto a^{-1}c^{-t},$$

where, again, the twist comes from the action on *xy^t^*. We deduce that $p^{-1}U_{p}^{\text{naive}}=p^{-1}(p^{k}U_{p})=p^{t}U_{p}$ is matched with $\left[N({\mathbb{Z}}_{p})\text{diag}(p,1)N({\mathbb{Z}}_{p})\right]$, concluding the proof. □

Remark 2. Lemma 1 can also be deduced from ref. 6, theorem 1.0.1, and this has the advantage that it is stated already with completed cohomology instead of compactly supported completed cohomology and cusp forms. We have used ref. 5 above because it was easier to check carefully our own normalizations!

Remark 3. The vectors used for k≤0 also exist for k≥2, where they induce an injection on all overconvergent modular forms. The same argument then recovers the fact that *U_p_* has nonnegative slopes when k≥2 (of course, it is much simpler to deduce this from the action on *q* expansions!). Representation theoretically, this vector comes from a highest weight Verma module, which, when k≥2, admits an algebraic quotient; the classical forms are exactly those that factor through the algebraic quotient, and the vector we used above for the k≥2 case is the lower highest weight vector generating the kernel. This perspective is explained in ref. 5.

Remark 4. The reason one is led to use different normalizations depending on k≤0 or k≥2 is mostly explained by the form of the Hodge–Tate sequence

$$\text{Lie}E(1)\to T_{p}(E)⊗O_{Y}\to \omega_{E^{\vee }}.$$

Indeed, since we are using the double-coset operator for the *p*-integral matrix diag(p,1) acting on $V_{p}E$, to obtain an operator with nonnegative slopes, it is natural to use the equivariant structure from $\omega_{E^{\vee }}$ for *k* = 1 and from $\text{Lie}E=\omega_{E}^{-1}$ for *k*  =  – 1, and similarly for larger |k| by taking symmetric powers of $T_{p}(E)$. The equivariant structures differ by an absolute value of the determinant, which manifests here as different powers of *p* for the double-coset operator coming from diag(p,1).

## Data Availability

There are no data underlying this work.

## Footnotes

^*^In ref. 1 and the introduction of ref. 2, this result is stated at level $\Gamma_{0}(p)$ using the smaller group of cohomology with support in $\bar{X_{0}{\left(p\right)}_{w}^{\text{ord}}}$. It is immediate from the results of *loco citato* that this smaller space has the same finite slope part, and arbitrary level is treated in ref. 2, theorem 5.12.2.
